# Proteomic identification and validation of novel interactions of the putative tumor suppressor PRELP with membrane proteins including IGFI-R and p75NTR

**DOI:** 10.1016/j.jbc.2021.100278

**Published:** 2021-01-09

**Authors:** Hirofumi Kosuge, Makoto Nakakido, Satoru Nagatoishi, Tetsuya Fukuda, Yasuhiko Bando, Shin-ichi Ohnuma, Kouhei Tsumoto

**Affiliations:** 1 School of Engineering, The University of Tokyo, Tokyo, Japan; 2 The Institute of Medical Science, The University of Tokyo, Tokyo, Japan; 3 Biosys Technologies, Tokyo, Japan; 4 The Institute of Ophthalmology, University College London, London, United Kingdom

**Keywords:** PRELP, small leucine-rich proteoglycan (SLRP), protein–protein interaction, proteomics, membrane protein, growth factor receptor, coimmunoprecipitation, surface plasmon resonance (SPR), CoIP-MS, coimmunoprecipitation coupled with mass spectrometry, DDM, n-dodecyl-β-D-maltoside, ESI, electrospray ionization, GO, gene ontology, IGFI-R, insulin-like growth factor I receptor, IGFs, insulin-like growth factors, *K*_D_, dissociation constant, LRP1, low-density lipoprotein receptor-related protein 1, LRR, leucine-rich repeat, mAb, monoclonal antibody, NF-κB, nuclear factor-kappa B, NGF, nerve growth factor, P1, passage 1, P2, passage 2, P3, passage 3, p75NTR, low-affinity nerve growth factor receptor p75NTR, PRELP, proline and arginine-rich end leucine-rich repeat protein, rPRELP, recombinant PRELP, rsIGFI-R, recombinant, soluble IGFI-R, rsp75NTR, recombinant, soluble p75NTR, SLRPs, small leucine-rich repeat proteoglycans, SPR, surface plasmon resonance, TGF-β, transforming growth factor-β

## Abstract

Proline and arginine-rich end leucine-rich repeat protein (PRELP) is a member of the small leucine-rich repeat proteoglycans (SLRPs) family. Levels of *PRELP* mRNA are downregulated in many types of cancer, and PRELP has been reported to have suppressive effects on tumor cell growth, although the molecular mechanism has yet to be fully elucidated. Given that other SLRPs regulate signaling pathways through interactions with various membrane proteins, we reasoned that PRELP likely interacts with membrane proteins to maintain cellular homeostasis. To identify membrane proteins that interact with PRELP, we carried out coimmunoprecipitation coupled with mass spectrometry (CoIP-MS). We prepared membrane fractions from Expi293 cells transfected to overexpress FLAG-tagged PRELP or control cells and analyzed samples precipitated with anti-FLAG antibody by mass spectrometry. Comparison of membrane proteins in each sample identified several that seem to interact with PRELP; among them, we noted two growth factor receptors, insulin-like growth factor I receptor (IGFI-R) and low-affinity nerve growth factor receptor (p75NTR), interactions with which might help to explain PRELP’s links to cancer. We demonstrated that PRELP directly binds to extracellular domains of these two growth factor receptors with low micromolar affinities by surface plasmon resonance analysis using recombinant proteins. Furthermore, cell-based analysis using recombinant PRELP protein showed that PRELP suppressed cell growth and affected cell morphology of A549 lung carcinoma cells, also at micromolar concentration. These results suggest that PRELP regulates cellular functions through interactions with IGFI-R and p75NTR and provide a broader set of candidate partners for further exploration.

Protein–protein interactions are crucial for biological functions. Membrane proteins are particularly important as they mediate cellular responses to the environment (1, 2). Membrane proteins are also crucial drug targets (1). Due to the significance of the interactions of membrane proteins with other factors, a number of approaches have been developed to analyze the interactomes of membrane proteins (2, 3, 4, 5).

The small leucine-rich repeat proteoglycans (SLRPs) are a family of 17 secreted proteins (6, 7, 8). Each SLRP appears to interact specifically with multiple secreted and membrane proteins, regulating the signaling pathways and thereby cellular responses (6, 7, 8, 9, 10, 11, 12, 13, 14, 15). For example, the SLRP decorin interacts with several growth factor receptors including epidermal growth factor receptor, insulin-like growth factor I receptor (IGFI-R), c-Met, and vascular endothelial growth factor receptor 2. These interactions cause up- or downregulation of the downstream signaling pathways, leading to the modulation of cell proliferation, survival, adhesion, migration, and invasion (6, 7, 8, 9, 10, 11, 14, 15). Another SLRP, Tsukushi, is an antagonist of the Wnt signaling pathway by binding to Frizzled4, competing with Wnt2b and thus prevents Wnt activation through β-catenin-dependent signaling. The inhibition of Wnt signaling by Tsukushi leads to the regulation of retinal and peripheral eye development (6, 12).

Proline and arginine-rich end leucine-rich repeat protein (PRELP) is a member of the SLRPs family. Like several other SLRPs, PRELP is known to bind to type I and II collagen fibrils *via* leucine-rich repeat (LRR) domain (16, 17), and the unique N-terminal domain of PRELP interacts with heparin and heparan sulfate (16, 18). PRELP also interacts with several proteins involved in the complement system including C4b-binding protein, C3, and C9. The interaction of PRELP with C9 blocks C9 polymerization, which inhibits formation of the complement membrane attack complex (19). Furthermore, it has been recently revealed that PRELP interacts with a growth factor, transforming growth factor-β1 (TGF-β1), as with many other SLRPs, resulting in the suppression of Smad2 phosphorylation (20). Although PRELP-mediated inhibition of TGF-β pathway was previously reported (20), it was yet to be understood whether PRELP interacts with any growth factor receptors. Analysis of the ONCOMINE database showed that gene expression of *PRELP* is suppressed in many types of cancer (21). In addition, recent studies have indicated that PRELP has functions as a tumor-suppressing protein inhibiting the progression of bladder cancer (20) and hepatocellular carcinoma (22). It was also reported that in a mouse model the N-terminal peptide of human PRELP inhibits breast tumor cell growth and metastasis, contributing to a reduction in cachexia and increased survival (23). These data suggest that PRELP is likely to be essential for maintenance of cellular homeostasis.

Given that other SLRPs regulate cellular functions through modulation of signaling pathways *via* interactions with membrane proteins, we hypothesized that PRELP also interacts with multiple membrane proteins and regulates signaling pathways. To address this hypothesis, we used a proteomic approach to identify membrane proteins that interact with PRELP. To identify novel membrane proteins that interact with PRELP, we used coimmunoprecipitation coupled with mass spectrometric analysis (CoIP-MS). The protocol included use of an S-Trap column, which effectively removes detergents from samples. We identified various membrane proteins, including two growth factor receptors, IGFI-R and low-affinity nerve growth factor receptor (p75NTR), as interacting partners. We validated that PRELP binds directly to the extracellular domains of these two receptors by analyses of recombinant proteins using surface plasmon resonance (SPR) analysis. In addition, we showed that the addition of micromolar concentration of recombinant PRELP to A549 lung carcinoma cells induced the suppression of cell growth and change of cell morphology. Our investigation furthers our understanding of PRELP functions at the molecular level.

## Results

### Mass spectrometric identification of the proteins that interact with PRELP

**Figure 1:**
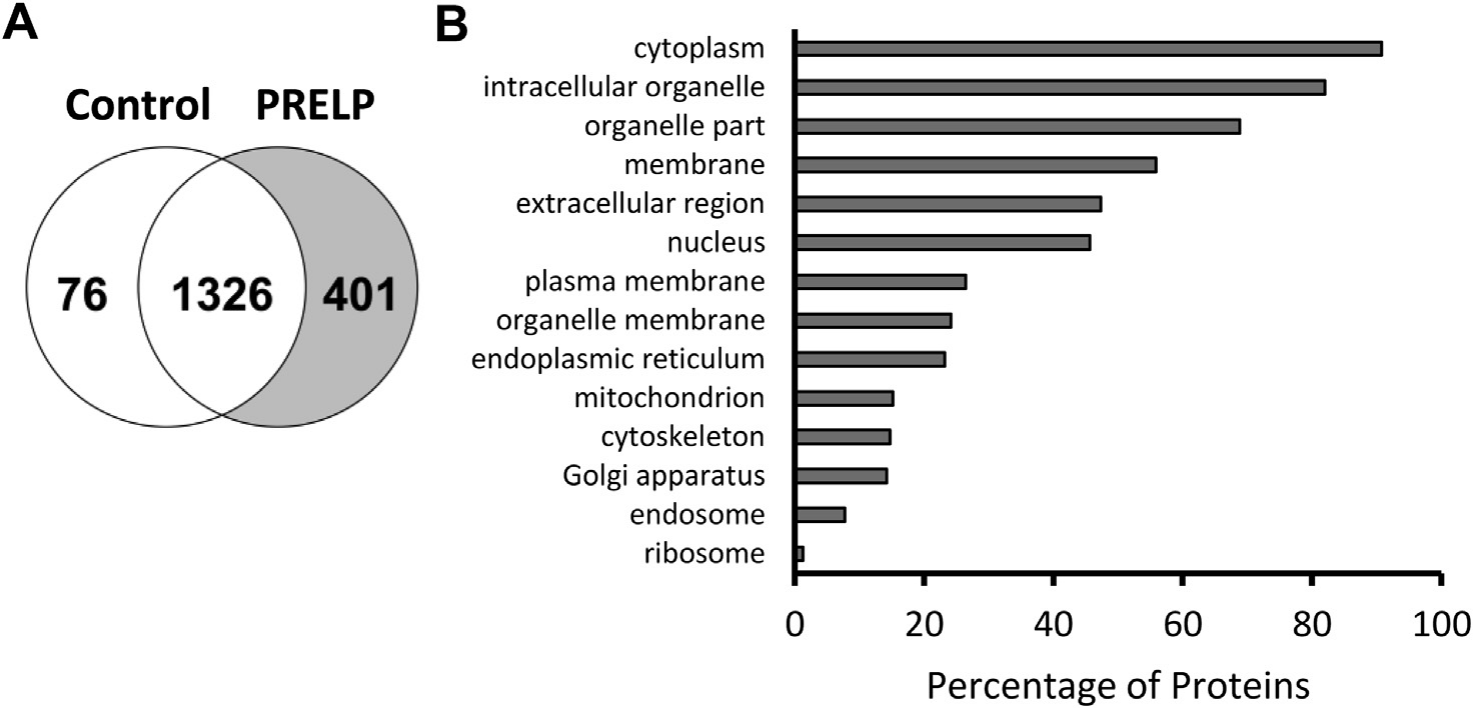
**Mass spectrometric identification and classification of proteins precipitated with PRELP.***A*, a Venn diagram of proteins detected in control samples and samples from PRELP-expressing cells by CoIP-MS (protein threshold: 99%; minimum number of unique peptides: 2; peptide threshold: 95%). *B*, percentage of proteins shown to interact with PRELP with indicated GO cellular component terms.

To identify the membrane proteins that interact with PRELP, we performed CoIP-MS. Human PRELP with a FLAG-tag was overexpressed in Expi293 cells, a human cell line in which proteins can be expressed at high levels. Membrane fractions from cells transfected with control vector and the PRELP expression vector were isolated by ultracentrifugation from sonicated cells. Membrane proteins were extracted using the detergent n-dodecyl-β-D-maltoside (DDM). An antibody that recognizes the FLAG-tag of the expressed PRELP was used to capture PRELP and associated proteins. Subsequent MS analysis of eluted samples identified 1803 proteins (protein threshold: 99%; minimum number of unique peptides: 2; peptide threshold: 95%). All assigned peptides are listed in Table S1. Comparison of identified proteins in each sample showed that 1326 proteins were found in samples from both control and PRELP-expressing cells and that 401 different proteins were identified only in the PRELP-expressing cells sample (Fig. 1*A* and Table S2). In total, 76 proteins were found only in the control cells sample, for the expression of these proteins might be downregulated in the PRELP-expressing cells.

To assess the localization of the proteins that appear to interact with PRELP, we used gene ontology (GO) analysis. A number of proteins that interact with PRELP localize in membrane fractions, although the analysis also suggested that a large portion of the proteins identified are localized intracellularly (Fig. 1*B*). Plasma membrane proteins, defined as proteins that have both extracellular domains and transmembrane domains spanning the plasma membrane, that interact with PRELP were identified by a search of the Uniprot database (24). We identified 29 plasma membrane proteins that would interact with PRELP (Table 1).

**Table 1:** Membrane proteins that interact with PRELP as shown by CoIP-MS^a^

Accession number (Uniprot_ID)	Protein name	Peptide count		Percent Coverage	
		n1	n2	n1	n2
1A02_HUMAN	HLA class I histocompatibility antigen, A-2 alpha chain	4	8	15.9%	29.9%
AAAT_HUMAN	Neutral amino acid transporter B(0)	3	5	6.7%	10.9%
ADA15_HUMAN	Disintegrin and metalloproteinase domain-containing protein 15	2	1	2.9%	1.9%
AGRL2_HUMAN	Adhesion G-protein-coupled receptor L2	3	4	3.6%	4.7%
AT2B1_HUMAN	Plasma membrane calcium-transporting ATPase 1	3	3	3.0%	3.8%
CBPD_HUMAN	Carboxypeptidase D	2	5	1.7%	4.4%
CD97_HUMAN	CD97 antigen	3	3	6.1%	6.1%
CDHR1_HUMAN	Cadherin-related family member 1	2	2	4.0%	5.0%
CELR2_HUMAN	Cadherin EGF LAG seven-pass G-type receptor 2	2	0	1.0%	0.0%
CNNM4_HUMAN	Metal transporter CNNM4	0	2	0.0%	2.5%
CSPG4_HUMAN	Chondroitin sulfate proteoglycan 4	3	4	1.9%	2.4%
CSTN1_HUMAN	Calsyntenin-1	2	3	2.9%	4.2%
CSTN3_HUMAN	Calsyntenin-3	6	7	12.6%	12.7%
DSG2_HUMAN	Desmoglein-2	9	9	12.4%	13.0%
FAT1_HUMAN	Protocadherin Fat 1	53	42	16.6%	12.6%
FRAS1_HUMAN	Extracellular matrix protein FRAS1	18	19	6.7%	7.3%
FREM2_HUMAN	FRAS1-related extracellular matrix protein 2	47	49	20.5%	20.3%
IGF1R_HUMAN	Insulin-like growth factor 1 receptor	7	5	6.5%	5.1%
ITM2B_HUMAN	Integral membrane protein 2B	2	0	8.7%	0.0%
LRP1_HUMAN	Prolow-density lipoprotein receptor-related protein 1	6	4	1.8%	1.2%
PCD19_HUMAN	Protocadherin-19	1	2	0.9%	2.8%
PCD20_HUMAN	Protocadherin-20	7	7	10.1%	10.1%
PCDH7_HUMAN	Protocadherin-7	3	3	3.8%	3.8%
PCSK5_HUMAN	Proprotein convertase subtilisin/kexin type 5	2	1	1.6%	0.7%
RENR_HUMAN	Renin receptor	2	3	6.6%	8.9%
TFR1_HUMAN	Transferrin receptor protein 1	7	8	11.1%	13.0%
TMED1_HUMAN	Transmembrane emp24 domain-containing protein 1	1	2	4.0%	9.7%
TMX1_HUMAN	Thioredoxin-related transmembrane protein 1	4	3	12.1%	8.6%
TMX4_HUMAN	Thioredoxin-related transmembrane protein 4	2	1	7.2%	3.7%

a Selection criteria were protein threshold: 99%; minimum number of unique peptides: 2; peptide threshold: 95%. Samples were analyzed twice using different injection volumes: n1 samples: 1 μl; n2 samples: 5 μl. All proteins present in the CoIP of PRELP-expressing cells are listed in Table S2.

### Growth factor receptors IGFI-R and p75NTR interact with PRELP

**Figure 2:**
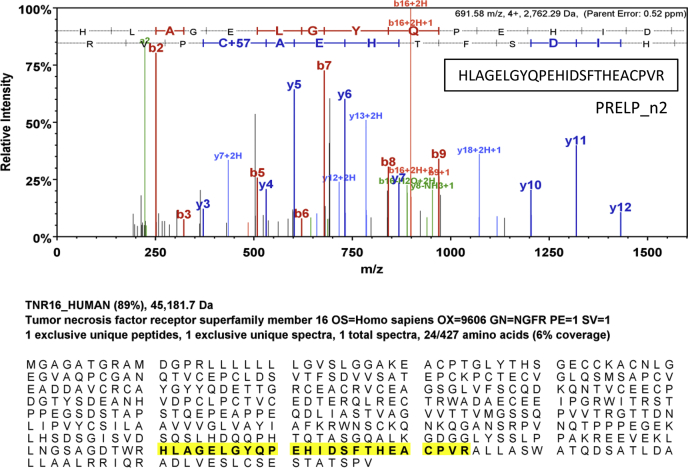
**Mass spectrometric identification of p75NTR as a PRELP interactor.** MS/MS spectra of the peptide assigned to p75NTR (TNR16_HUMAN) and the location of the peptide within the p75NTR amino acid sequence. Details of peptide assignments are presented as Table S3.

**Figure 3:**
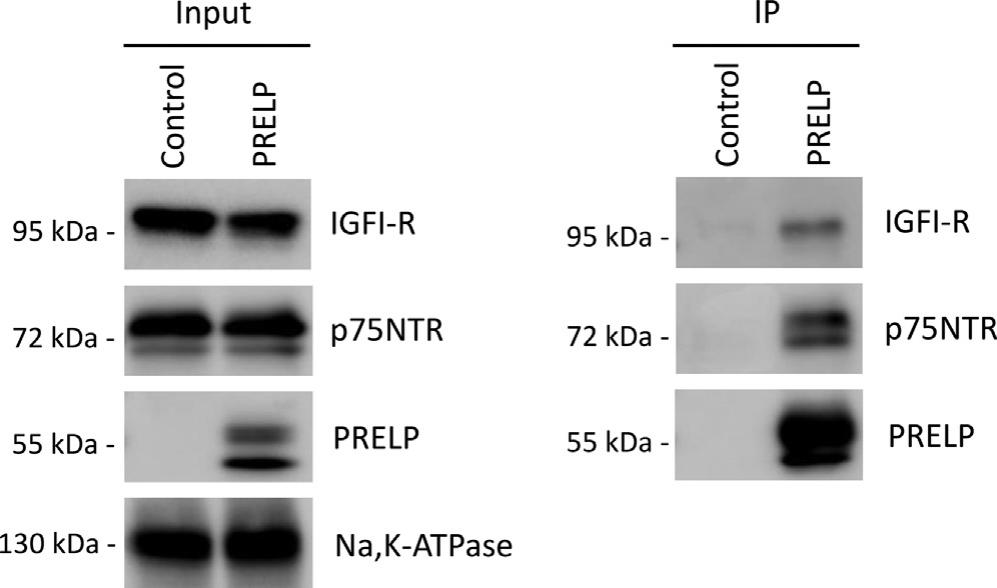
**Western blot analysis validates the interactions of PRELP with IGFI-R and p75NTR.** Samples before (left) and after (right) immunoprecipitation of samples from control and PRELP-expressing cells were subjected to SDS-PAGE and transferred to membranes. PRELP, IGFI-R, p75NTR, and Na,K-ATPase were detected by monoclonal antibodies.

Many SLRPs interact with various growth factors and their receptors, and these interactions are thought to be critical for the functions of the SLRPs (6, 7, 8, 9, 10, 11, 12, 13, 14, 15). More recently, PRELP and osteomodulin, a most highly conserved SLRP member with PRELP, have been also found that they regulate bladder cancer initiation in a functionally partially redundant manner. The study showed that osteomodulin inhibits the pathways of two growth factors, TGF-β and epidermal growth factor (20), while the growth factor receptors targeted by PRELP were not clear. Therefore, we particularly focused on growth factor receptors coprecipitated with PRELP, which would be key proteins to explain molecular function of PRELP as tumor suppressor. Among the plasma membrane proteins identified by the mass spectrometric analysis was IGFI-R, a receptor known to interact with the SLRPs decorin and asporin (6, 7, 8, 11, 13, 14, 15). In addition, the low-affinity nerve growth factor receptor known as p75NTR was detected. Although only one peptide fragment of p75NTR was assigned under the 80% of protein threshold and 95% of peptide threshold, the score of MS/MS spectra of this peptide was high, and this peptide sequence is unique to this protein (Fig. 2 and Table S3). To validate the interactions of PRELP with these two receptors, we carried out CoIP using the antibody that recognizes the FLAG-tag of the expressed PRELP followed by western blot analysis. Both IGFI-R and p75NTR were detected in CoIP-eluates of extracts of cells that express PRELP but not in control-transfected Expi293 cells (Fig. 3). Incidentally, two bands of western blot observed in PRELP and p75NTR are probably due to the heterogeneity of glycan modification. These experiments indicate that these two receptors specifically interact with PRELP.

### PRELP interacts with the extracellular domains of p75NTR and IGFI-R

**Figure 4:**
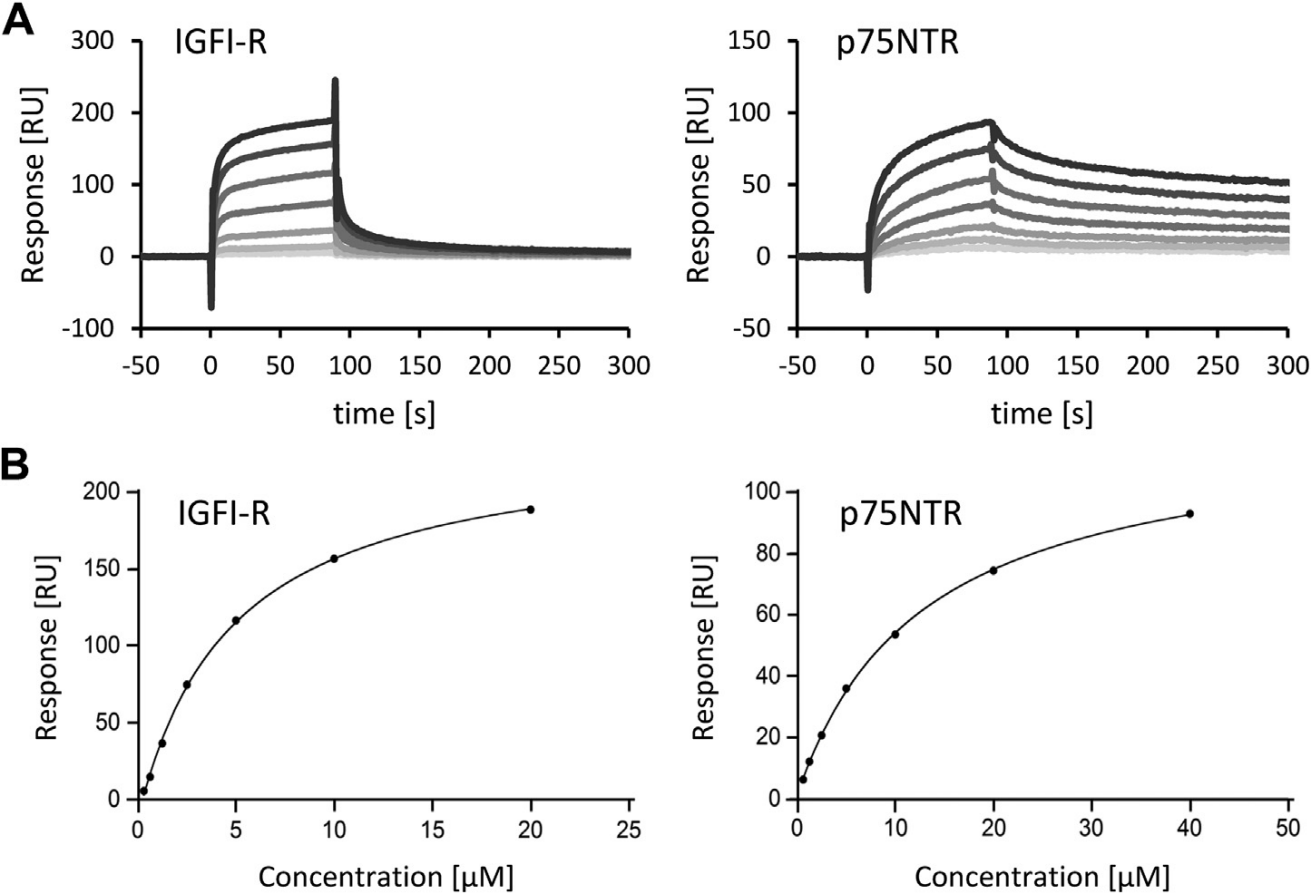
**PRELP interacts with the extracellular domains of IGFI-R and p75NTR.***A*, SPR response vs. time curves for interaction of rsIGFI-R (left) and rsp75NTR (right) with rPRELP at 15 °C. Line darkness indicates concentration: The darkest line indicates the highest concentration. *B*, response units vs. concentration of rsIGFI-R (left) and rsp75NTR (right). Three independent measurements were carried out, and the average values of *K*_D_ with standard errors were calculated: *K*_D_ of rsIGFI-R = 6.8 ± 2.4 μM and *K*_D_ of rsp75NTR = 12.1 ± 2.0 μM.

The SLRPs are evaluated as secreted proteins (6, 7, 8), and it is likely that PRELP is also secreted. We therefore expected that PRELP interacts with the extracellular domains of IGFI-R and p75NTR. To confirm this, we conducted SPR analysis using recombinant proteins. We expressed and purified the extracellular domains of IGFI-R and p75NTR and evaluated the interactions with recombinant PRELP (rPRELP). SPR analysis demonstrated that PRELP binds to the extracellular domains of both IGFI-R and p75NTR (Fig. 4*A*). Dissociation constants (*K*_D_) were calculated by fitting the equilibrium curve (Fig. 4*B*). Recombinant, soluble IGFI-R (rsIGFI-R) and p75NTR (rsp75NTR) showed similar and relatively low affinities for rPRELP (rsIGFI-R: *K*_D_ = 6.8 ± 2.4 μM; rsp75NTR: *K*_D_ = 12.1 ± 2.0 μM). It is worth noting that the shapes of SPR sensorgrams for binding to IGFI-R and p75NTR were significantly different. rsIGFI-R bound to rPRELP with faster association and dissociation rates than rsp75NTR (Fig. 4*A*). This result suggests that the mechanism of how PRELP interacts with IGFI-R is different from that with p75NTR.

### PRELP suppresses cell growth and changes cell morphology in the micromolar range

**Figure 5:**
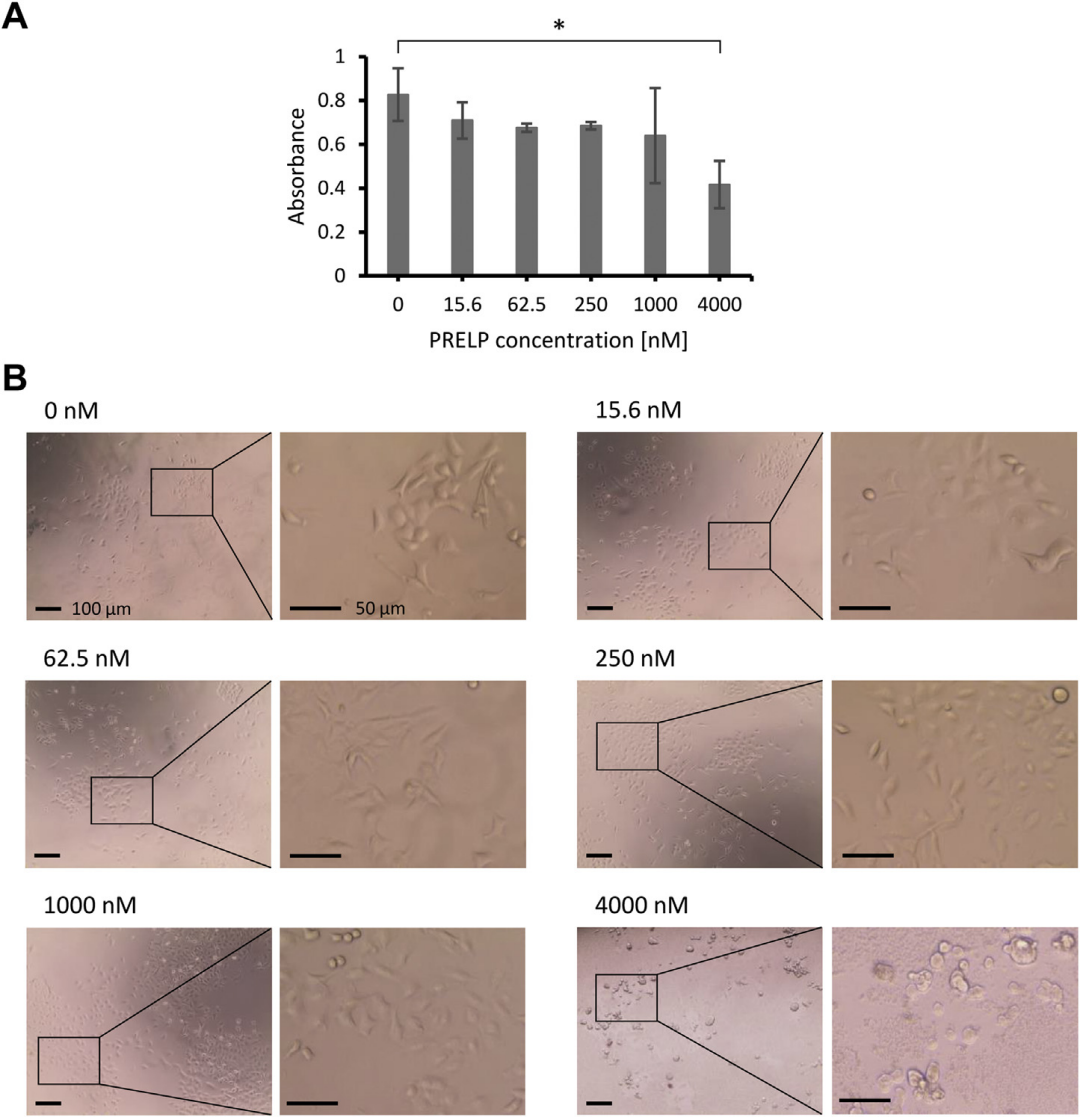
**PRELP suppresses growth and changes morphology of A549 lung carcinoma cells.***A*, cell growth ability of A549 cells treated with different concentration of rPRELP was evaluated using Cell Count Reagent SF. The absorbance at 450 nm is proportional to the number of viable cells 96 h after the addition of rPRELP. The average values with standard deviations of independent three wells are shown as bar chart. ∗ indicates *p* < 0.05. *B*, The morphology of A549 cells 96 h after the treatment of different concentration of rPRELP was observed by microscopy. Scale bar represents 100 μm (each left) and 50 μm (each right).

SPR analysis validated the relatively low-affinity interaction of PRELP with IGFI-R and p75NTR by the micromolar range of *K*_D_ values. Although the SLRP decorin also interacts with IGFI-R, the affinity was reported to be in the nanomolar range (14, 15). To assess the physiological relevance of these weak micromolar interactions, we evaluated the quantitative influence of different concentrations of rPRELP on tumor cell proliferation and morphology, which have been qualitatively validated in previous studies (20, 22). For cell-based assay, we used a human lung carcinoma cell line A549, as gene expression level of *PRELP* is suppressed in the majority of lung cancer types (21). We firstly evaluated the growth activity of A549 cells treated with rPRELP in the concentration-dependent manner (Fig. 5*A*). The cell growth assay demonstrated that 4 μM of rPRELP significantly suppressed the cell growth, whereas lower concentration of rPRELP did not show significant suppression. Next, we evaluated the morphology of cells treated with rPRELP (Fig. 5*B*). The addition of rPRELP resulted in a change of cell morphology to round shapes, as observed by overexpression of PRELP (20). This change of cell morphology was observed only in the micromolar range, namely low concentration of PRELP appears not to affect the morphology of A549 cells. Although it remains unclear whether these phenomena were triggered *via* the interactions of PRELP with IGFI-R, p75NTR, or other membrane proteins that we identified, our results indicate that PRELP functions in the micromolar range of concentration, which is consistent with the micromolar affinities of the interaction of PRELP with IGFI-R and p75NTR.

## Discussion

In this study, we identified membrane proteins that interact with the glycoprotein PRELP using a proteomic approach. In general, proteomic analyses of membrane proteins are difficult because these proteins are hydrophobic and poorly soluble (2). Although detergents are needed to extract and solubilize membrane proteins, detergents tend to unfold proteins and diminish protein–protein interactions. Instead of using a strong detergent for cell lysis, we isolated the membrane fraction from physically disrupted cells by sonication followed by ultracentrifugation, and membrane proteins were extracted with DDM, a gentle detergent. In addition, detergents can inhibit enzymatic digestion necessary for mass spectrometric analysis. In-solution removal of detergent is challenging, so we used an S-Trap column, recently developed for efficient sample preparation (5) to rapidly prepare membrane protein extracts free of detergent. We also performed protein digestion and peptide cleanup on the same column. Relying on these techniques, we identified membrane proteins that specifically bind to PRELP. As different detergents solubilize different types of membrane proteins (25), using detergents other than DDM may enable identification of other protein partners of PRELP.

In this research, we validated the interactions of PRELP with two growth factor receptors, IGFI-R and p75NTR. IGFI-R is a type I receptor of insulin-like growth factors (IGFs), which is widely expressed in normal and tumor tissues, and both the growth factors and the receptor are attractive targets for cancer therapy (26, 27, 28, 29, 30, 31). Indeed, anti-IGFI-R antibodies have been evaluated clinically, although results have been mixed (26, 29, 31). The SLRP decorin binds to IGFI-R with nanomolar range of affinity, resulting in both positive and negative regulation of IGFI-R downstream signaling pathway in the cell-type-dependent manner (14, 15). Given that PRELP binds to IGFI-R with the micromolar affinity, the binding mechanism to IGFI-R and following functions are likely to be different between PRELP and decorin.

There have been no reports of interactions of any SLRPs with p75NTR. p75NTR is a receptor for neurotrophins such as nerve growth factor (NGF), a key nervous system growth factor. p75NTR and the selective tyrosine kinase receptor TrkA transmit NGF-induced signals that mediate proliferation, differentiation, and survival of neurons (32, 33, 34). In addition, these two receptors of NGF are implicated in cancer development (35, 36, 37). p75NTR has been reported to have antiapoptotic function in breast cancer cells, through the activation of signaling mediated by nuclear factor-kappa B (NF-κB) (35, 36). These data suggest that PRELP modulates signaling involving two growth factor receptors and therefore is likely involved in maintenance of cellular homeostasis and suppression of tumor cell growth.

In addition to these two growth factor receptors, we identified a number of other membrane proteins that would interact with PRELP (Table 1), including low-density lipoprotein receptor-related protein 1 (LRP1). LRP1 is also known to interact with decorin, resulting in the modulation of TGF-β mediated signaling pathway (38, 39). As LRP1 is also known to negatively regulate the canonical Wnt signaling pathway through the interaction with Frizzled-1 receptor (40), PRELP may be involved in the Wnt signaling pathway just as the SLRP Tsukushi (12). Our proteomic analysis showed that PRELP also interacts with six nonclassical cadherins including four nonclustered protocadherins (Protocadherin-7, Protocadherin-19, Protocadherin-20, and Cadherin-related family member 1 also known as Protocadherin-21) and three adhesion G-protein-coupled receptors (Adhesion G-protein-coupled receptor L2, CD97 antigen, and Cadherin EGF LAG seven-pass G-type receptor 2). Protocadherins have diverse functions as mediators of cell adhesion and cell motility that are involved in brain development, neurological disorders, and tumorigenesis (41, 42). Adhesion G-protein-coupled receptors are widely expressed in normal and malignant tissues and appear to mediate the movement of neurons, leukocytes, and tumor cells through the regulation of cell–cell and cell–matrix interactions (43, 44). Further, we also applied to GO analysis in terms of biological processes (Table S4), showing that 12 out of 29 membrane proteins that interact with PRELP are also involved in biological adhesion. These observations suggest that PRELP regulates cell attachment and motility *via* the interactions with these proteins. Several previous studies also demonstrated the contributions of PRELP to cell adhesion (20, 22, 45). The overexpression of PRELP results in the upregulation of gene expression of cell–cell tight junction components, indicating that PRELP positively regulates cell–cell adhesion (20). It was also reported that the formation of fibroblast focal adhesion is enhanced by N-terminal domain of PRELP together with the integrin-binding part of fibronectin (45).

GO cellular component term analysis suggested that in addition to membrane-bound proteins, PRELP also interacts with cytoplasmic proteins and proteins localized to organelles (Fig. 1*B*). There is a possibility that these intracellular proteins functionally interact with PRELP. Especially, the mass spectrometric identification profiles showed a large number of different enzymes including various degrading enzymes, transferases, isomerases, and kinases (Table S2), suggesting a possibility that PRELP might also relate to the regulation of these enzymatic activities. Intriguingly, nuclear-localized proteins constitute a large proportion of the identified interactors. For example, several nuclear-localized proteins involved in the regulation of NF-κB signaling or NF-κB binding such as CDK5 regulatory subunit-associated protein 3, copine-1, catenin beta-1, and signal transducer and activator of transcription 1-alpha/beta were identified in our analysis (Table S2). This suggests that PRELP might also localize to and function in the nucleus, though SLRPs are generally localized in the extracellular region. In support of this hypothesis, previous studies showed that PRELP is localized in the nucleus as well as cytoplasm of hepatocellular carcinoma tissues (22), and the N-terminal peptide of PRELP is also translocated to the nucleus where it inhibits the DNA-binding activity of NF-κB (46). In addition, it was reported that the overexpression and depletion of PRELP significantly affect the gene expression profiles of NF-κB pathway (20).

As above, our proteomic analysis indicated that PRELP is involved in various functions through the interactions with multiple proteins. LRR proteins such as Toll-like receptors are well known to interact with a wide variety of ligands *via* the LRR domain (47, 48), and LRR domain of PRELP also has a binding ability to collagen fibrils (16). In addition to LRR domain, PRELP has the positively charged N-terminal region binding to different molecules including glycosaminoglycan chains and p65 NF-κB (16, 18, 46). This unique constitution of PRELP containing both LRR domain and N-terminal region might lead to the wide range of binding properties of PRELP.

Our cell-based assay using A549 lung carcinoma cells demonstrated that PRELP suppressed the cell growth and changed the cell morphology in the micromolar range. Since we added recombinant PRELP instead of overexpression, these phenomena should not attribute to the interactions of PRELP with intracellular proteins but to those with extracellular or cell surface proteins. Considering the micromolar efficacy, PRELP possibly affects the growth and morphology of A549 cells, triggered by the regulation of signaling pathways or cell adhesion through relatively weak but meaningful interactions with membrane proteins that we identified in this study. Of note, PRELP is also known to strongly interact with some basement membrane components such as a proteoglycan perlecan *via* its heparan sulfate chains (16). To observe the influence of low-affinity interactions of PRELP and exclude the influence of strong binding to glycosaminoglycan chains of cell-surface proteoglycans, we selected A549 lung carcinoma cells, since it has been previously reported that PRELP was not attached to A549 cell surface probably due to the different sulfation of glycosaminoglycans (45). Our results strongly suggest that the low-affinity interactions of PRELP are important for the biological functions as well as the high-affinity interactions. Although it is unclear whether *in vivo* concentration of PRELP reaches to the micromolar range, anchoring of PRELP to the components of basement membrane and extracellular matrix such as collagen may increase the local concentration of PRELP in the adjacent area of cell surface.

In conclusion, we identified novel membrane proteins, including two growth factor receptors IGFI-R and p75NTR, as well as nuclear and cytoplasmic factors that interact with the putative tumor repressor protein PRELP using a CoIP-MS strategy. The functions of IGFI-R and p75NTR are diverse, and the interactions with these factors are likely to mediate some of the biological functions of PRELP. Although many of the interactions we identified remain to be validated, our investigation is a breakthrough in the elucidation of the biological functions of PRELP.

## Experimental procedures

### Transfection and expression of PRELP in Expi293 cells

The DNA sequence encoding human PRELP with a FLAG tag at the C terminus was subcloned into the pcDNA3.4 vector (Thermo Fisher Scientiﬁc). The DNA sequence of the signal peptide region of human PRELP with a FLAG tag at the C terminus was also subcloned into the pcDNA3.4 vector and was used as a control. Expi293 cells (Thermo Fisher Scientiﬁc) were transfected with resultant vectors using the ExpiFectamine 293 Transfection Kit (Thermo Fisher Scientiﬁc) in accordance with the manufacturer’s protocol. The cells were cultured for 2–3 days at 37 °C and 8% CO_2_.

### Preparation of membrane fraction samples

Cells were collected, and membranes were disrupted using an ultrasonic cell-disrupting UD-201 instrument (TOMY). Centrifugation at 1000 × *g* for 10 min at 4 °C removed debris. The supernatant was centrifuged at 210,000 × *g* for 90 min at 4 °C, and membrane fraction was collected. The membrane fraction was homogenized in PBS (pH 7.4) containing 2% (w/v) DDM and rotated for 30 min at 4 °C. The sample was centrifuged at 210,000 × *g* for 30 min at 4 °C. The supernatant was collected, and the total protein concentration was measured with a BCA protein assay kit (Thermo Fisher Scientiﬁc) using bovine serum albumin as a standard.

### Coimmunoprecipitation

After the adjustment of the total protein concentration to 4 mg/ml, the samples containing membrane proteins were diluted 20-fold with PBS (pH 7.4) to adjust the final concentration of DDM to 0.1% (w/v). DDDDK-tagged Protein Purification Gel (MBL) was added to each sample, and samples were rotated overnight at 4 °C. The gel was collected by centrifugation and washed three times with PBS (pH 7.4) containing 0.1% (w/v) DDM. The proteins were then eluted with PBS (pH 7.4) containing 0.1% (w/v) DDM and 1 mg/ml FLAG peptide (Sigma Aldrich).

### Sample preparation for mass spectrometry

The membrane protein samples were reduced by treatment with 20 mM dithiothreitol at 95 °C for 10 min and then alkylated with 40 mM 2-iodoacetamide shielded from light at room temperature for 30 min. Aqueous phosphoric acid was added to each sample to a final concentration 1.2%, and S-Trap buffer (90% aqueous methanol containing 100 mM tetraethylammonium bromide (pH 7.1)) was added to the sample at sixfold the sample volume. The samples were then applied to an S-Trap micro column (ProtiFi). The column was centrifuged to capture proteins within the spin column and was then washed with 150 μl of S-Trap buffer. Trapped proteins were digested with trypsin in the column at 47 °C for 60 min. Firstly, nonhydrophobic digested peptides were eluted with 40 μl of 50 mM tetraethylammonium bromide and then 40 μl of 0.2% aqueous formic acid. Finally, hydrophobic peptides were recovered by elution with 35 μl of 50% acetonitrile containing 0.2% formic acid. These eluates were combined, dried under vacuum, and then resuspended in 100 μl of 2% acetonitrile containing 0.1% formic acid.

### LC-MS/MS

The processed samples were subjected to nanoflow reversed-phase LC followed by tandem MS using a Q-Exactive mass spectrometer (Thermo Fisher Scientific) equipped with a Dream Spray electrospray ionization (ESI) source (AMR Inc) and XYZ stage (AMR Inc) as previously described (49, 50, 51). A capillary reverse-phase HPLC–MS/MS system consists of an Ultimate 3000 dual-solvent delivery system (Thermo Fisher Scientific) and a PAL LSI auto-sampler (CTC Analytics).

To desalinate and concentrate the peptides, the sample solutions were automatically injected into a peptide LC precolumn (L-column ODS, Micro C18, 5 × 0.3 mm; Chemical Evaluation Research Institute) attached to an injector valve. Both control and PRELP samples were analyzed twice using different injection volumes (n1 samples: 1 μl; n2 samples: 5 μl). The samples were loaded onto a capillary reverse-phase separation column packed with 1.6-μm-diameter gel particles of pore size 120 Å (AURORA C18, 250 × 0.075 mm; IonOpticks). Eluent A was 0.1% formic acid, and eluent B was 100% acetonitrile. The gradient was A + 5% B to 35% B in 100 min and from 35% B to 95% B in 1 min, with subsequent isocratic elution with 95% B for 8 min and a further concentration gradient from 95% B to 5% B in 1 min. The flow rate column was 0.2 μl/min. The effluxes were introduced into the mass spectrometer through the ESI interface using a Dream Spray (AMR) with a separation column outlet connected directly to the ESI needle (AURORA C18, 250 × 0.075 mm, IonOpticks). The spray voltage was 1.4 kV.

The mass spectrometer was operated in a data-dependent acquisition mode in which the MS acquisition with a mass range of m/z 400–1600 was automatically switched to MS/MS acquisition under the automated control of Xcalibur software 3.1 (Thermo Fisher Scientific). MS scan was selected by Orbitrap, with resolution or 70,000. Subsequent MS/MS scans used an ion trap in automated gain control mode with values of 1 × 10^6^ and 1 × 10^4^ for full MS and MS/MS, respectively. For fragmentation, collision-induced dissociation was used. Ions of the full MS scan were identified by the Xcalibur software as precursor ions and were subjected to MS/MS with an isolation width of m/z 1.6 and a normalized collision energy parameter of 27. One MS and MS/MS cycles were set to 1.0 s. Selected ions were excluded from repeated sequencing by dynamic exclusion that was enabled with a repeat count of 2 over a duration 10 s and an exclusion window of 15 s and an exclusion mass width of 5 ppm (52).

### Data analysis

#### Database searching

The mass spectrometric raw data were converted into peaklist files using Xcalibur software 4.0.27.19 (Thermo Fisher Scientific) for database searching. Tandem mass spectra were extracted using Xcalibur software version 3.1 (Thermo Fisher Scientific). Charge-state deconvolution and deisotoping were not performed. All MS/MS samples were analyzed using Mascot (Matrix Science; version 2.6.1). Mascot was used to search the SwissProt database (selected for *Homo sapiens*, 20,418 entries, accessed 02/2019) assuming the digestion enzyme trypsin. The fragment ion mass tolerance was set to 0.025 Da, and the parent ion tolerance was set to 5.0 ppm. Carbamidomethyl of cysteine was specified in Mascot as a fixed modification. Dethiomethyl of methionine, gln→pyro-Glu of the N-terminus, oxidation of methionine, acetyl of the N-terminus, carbamidomethyl of methionine, and phosphorylation of serine, threonine, and tyrosine were specified as variable modifications.

#### Criteria for protein identification

Scaffold (version 4.10.0; Proteome Software) was used to validate MS/MS-based peptide and protein identifications. Peptide identifications were accepted if they were established at greater than 95.0% probability by the Peptide Prophet algorithm (53) with Scaffold delta-mass correction. Protein identifications were accepted if they were established at greater than 99.0% probability by the Protein Prophet algorithm and contained at least two identified peptides (54). Proteins that contained similar peptides that could not be differentiated based on MS/MS analysis alone were grouped to satisfy the principles of parsimony. Proteins were annotated with GO terms from NCBI (downloaded 2019/04/04) (55).

### Western blot analysis

The membrane fraction samples (input samples) and the samples obtained after CoIP were subjected to SDS-PAGE using a Tris-glycine gel. The samples were mixed with the loading buffer and 2-mercaptoethanol and heated at 95 °C for 5 min prior to loading. After electrophoretic separation, proteins were electrophoretically transferred onto PVDF membrane. The membrane was blocked with 5% (w/v) skim milk in 20 mM Tris-HCl (pH 7.4), 150 mM NaCl containing 0.1% (v/v) Tween-20 (TBS-T buffer) for 60 min. After washing the membrane with TBS-T, primary antibodies, diluted in TBS-T containing 5% (w/v) skim milk, were added, and the membrane was incubated overnight at 4 °C. Anti-DDDDK-tag mAb HRP-DirecT (MBL), which binds to the FLAG tag, was used to detect PRELP, IGF-I Receptor β rabbit mAb (Cell Signaling Technology) was used to detect IGFI-R, and p75NTR rabbit mAb (Cell Signaling Technology) was used to detect p75NTR. As a loading control for input samples, Na,K-ATPase was detected by Na,K-ATPase α1 rabbit mAb (Cell Signaling Technology). Anti-DDDDK-tag mAb HRP-DirecT was detected by the ECL detection system (Amersham) according to the manufacturer's instruction. The primary antibodies that recognize IGFI-R, p75NTR, and Na,K-ATPase were detected using anti-rabbit IgG HRP-linked antibody (Cell Signaling Technology) using the ECL detection system.

### Expression and purification of recombinant PRELP

The DNA sequence encoding human PRELP with a FLAG tag at the C terminus was subcloned into the pFASTBac1 vector (Invitrogen). Bacmids were prepared according to the manufacturer's protocol (Bac-to-Bac Baculovirus Expression System, Invitrogen). Sf9 insect cells (Thermo Fisher Scientific) were transfected with PRELP bacmids, followed by incubation at 27 °C for 4 days; the supernatant was passage 1 (P1) virus. Sf9 cells (1.0 × 10^6^ cells/ml) were infected with P1 virus (1:25, v/v) and incubated with shaking at 120 rpm at 27 °C for 2 days; the supernatant was passage 2 (P2) virus. The above procedure was repeated to prepare passage 3 (P3) virus. For rPRELP expression, 1.8 × 10^6^ cells/ml of Mimic Sf9 cells (Thermo Fisher Scientific) suspended in Sf900II serum-free medium (Thermo Fisher Scientific) containing 10% (v/v) fetal bovine serum were infected with P3 virus (1:50, v/v) and incubated with shaking at 120 rpm at 27 °C for 4–5 days. rPRELP was purified from the supernatant using DDDDK-tagged Protein Purification Gel (MBL). The gel was washed with PBS (pH 7.4), and protein was eluted with 1 M arginine-HCl (pH 4.4). The eluate was immediately neutralized with 2 M Tris-HCl (pH 8.0). The eluted fraction was treated with Benzonase nuclease (Millipore) as per the manufacturer’s protocol, and a second purification using DDDDK-tagged Protein Purification Gel was carried out. The eluate was purified by size-exclusion chromatography using a HiLoad 26/600 Superdex 200 pg column (GE Healthcare) equilibrated with 20 mM Tris-HCl (pH 8.0), 300 mM NaCl, 400 mM arginine-HCl.

### Expression and purification of the extracellular domain of IGFI-R

The DNA sequence encoding the extracellular domain of human IGFI-R (residues 1–905) with a hexahistidine tag at the C terminus was subcloned into the pcDNA3.4 vector. ExpiCHO cells (Thermo Fisher Scientiﬁc) were transiently transfected with the vector using ExpiFectamine CHO Transfection Kit (Thermo Fisher Scientiﬁc) in accordance with the manufacturer’s max-titer protocol. The cells were cultured for 2 weeks at 32 °C and 5% CO_2_. The recombinant, soluble IGFI-R (rsIGFI-R) was purified from the supernatant by a Ni-NTA agarose affinity column (Qiagen) equilibrated with binding buffer (20 mM Tris-HCl (pH 8.0), 150 mM NaCl, 5 mM imidazole). The column was ﬁrst washed with the binding buffer, and fractions were eluted with buffers containing increasing concentrations of imidazole (20–500 mM). The eluted fractions containing rsIGFI-R were further purified by size-exclusion chromatography using a HiLoad 26/600 Superdex 200 pg column (GE Healthcare) equilibrated with 20 mM Tris-HCl (pH 8.0), 150 mM NaCl.

### Expression and purification of the extracellular domain of p75NTR

The DNA sequence encoding the extracellular domain of human p75NTR (residues 1–161) with a hexahistidine tag at the C terminus was subcloned into the pFASTBac1 vector (Invitrogen). The extraction of bacmids and preparation of baculovirus were performed with the same procedure as used for rPRELP. For recombinant, soluble p75NTR (rsp75NTR) expression, 1.8 × 10^6^ cells/ml of Sf9 cells (Thermo Fisher Scientific) were infected with P3 virus (1:1000, v/v) and incubated with shaking at 120 rpm at 27 °C for 5 days. The rsp75NTR was purified from the supernatant by a Ni-NTA agarose affinity column (Qiagen) equilibrated with binding buffer (20 mM Tris-HCl (pH 8.0), 150 mM NaCl, 5 mM imidazole). The column was ﬁrst washed with the binding buffer, and fractions were eluted with buffers containing increasing concentrations of imidazole (20–500 mM). The eluted fractions containing the rsp75NTR fragment were further purified by size-exclusion chromatography using a HiLoad 26/600 Superdex 75 pg column (GE Healthcare) equilibrated with 20 mM Tris-HCl (pH 8.0), 150 mM NaCl.

### Surface plasmon resonance analysis

The interactions of rPRELP with rsIGFI-R and rsp75NTR were analyzed using SPR in a Biacore 2000 instrument (GE Healthcare). A CM5 Biacore sensor chip (GE Healthcare) was activated by treatment with N-hydroxysuccinimide/N-ethyl-N´-(3-dimethylaminopropyl) carbodiimide hydrochloride, followed by immobilization of rPRELP at around 4000 resonance units. After the immobilization, the activated surface of the sensor chip was blocked with 1 M ethanolamine hydrochloride (pH 8.5). rsp75NTR or rsIGFI-R was injected into the sensor chip at a ﬂow rate of 30 μl/min; a range of concentrations was tested. The association time was 90 s, and the dissociation time was 240 s. The assay was carried out in 10 mM HEPES (pH 7.5), 150 mM NaCl containing 0.05% (v/v) Tween-20 at 15 °C. A regeneration procedure was performed at the end of each cycle with 1 M arginine-HCl (pH 4.4). The data were analyzed with the BIAevaluation software (GE Healthcare), and the *K*_D_ was calculated by fitting the equilibrium curve using KaleidaGraph 4.5 software (HULINKS).

### Cell growth assay

Suspension of A549 cells (RIKEN) was seeded in 48-well plates for 2 × 10^3^ cells/well and preincubated in DMEM containing 10% (v/v) fetal bovine serum for 24 h at 37 °C and 5% CO_2_. Following the preincubation, the medium was replaced with 300 μl of new medium containing different concentrations of rPRELP. The cells were cultured for 96 h at 37 °C and 5% CO_2_. Then 100 μl of the medium containing 10% (v/v) Cell Count Reagent SF (Nacalai tesque) was added to each well and incubated for 2 h at 37 °C and 5% CO_2_. The absorbance at 450 nm of the supernatant was measured by PHERAstar micro plate reader (BMG LABTECH). The morphology of A549 cells was also observed with EVOS XL Core microscopy (Thermo Fisher Scientiﬁc) before the addition of Cell Count Reagent SF.

## Data Availability

The MS proteomics raw data and Scaffold file have been deposited to the Mendeley Data repository (https://data.mendeley.com/) with the data set identifier http://doi.org/10.17632/nmgrvjb9cw.1.

## Conflict of interest

The authors declare that they have no conflicts of interest with the contents of this article.

## References

[1] Yin H., Flynn A.D. (2016). Drugging membrane protein interactions. Annu. Rev. Biomed. Eng..

[2] Wu C.C., MacCoss M.J., Howell K.E., Yates J.R. (2003). A method for the comprehensive proteomic analysis of membrane proteins. Nat. Biotechnol..

[3] Borch J., Roepstorff P., Møller-Jensen J. (2011). Nanodisc-based co-immunoprecipitation for mass spectrometric identification of membrane-interacting proteins. Mol. Cell. Proteomics.

[4] Kamal A.H.M., Aloor J.J., Fessler M.B., Chowdhury S.M. (2019). Cross-linking proteomics indicates effects of Simvastatin on the TLR2 interactome and Reveals ACTR1A as a novel regulator of the TLR2 signal Cascade. Mol. Cell. Proteomics.

[5] Zougman A., Selby P.J., Banks R.E. (2014). Suspension trapping (STrap) sample preparation method for bottom-up proteomics analysis. Proteomics.

[6] Dellett M., Hu W., Papadaki V., Ohnuma S. (2012). Small leucine rich proteoglycan family regulates multiple signalling pathways in neural development and maintenance. Dev. Growth Differ..

[7] Schaefer L., Iozzo R.V. (2008). Biological functions of the small leucine-rich proteoglycans: From genetics to signal transduction. J. Biol. Chem..

[8] Pietraszek-Gremplewicz K., Karamanou K., Niang A., Dauchez M., Belloy N., Maquart F.X., Baud S., Brézillon S. (2019). Small leucine-rich proteoglycans and matrix metalloproteinase-14: Key partners?. Matrix Biol..

[9] Goldoni S., Humphries A., Nyström A., Sattar S., Owens R.T., McQuillan D.J., Ireton K., Iozzo R.V. (2009). Decorin is a novel antagonistic ligand of the Met receptor. J. Cell Biol..

[10] Khan G.A., Girish G.V., Lala N., di Guglielmo G.M., Lala P.K. (2011). Decorin is a novel VEGFR-2-binding antagonist for the human extravillous trophoblast. Mol. Endocrinol..

[11] Edwards I.J. (2012). Proteoglycans in prostate cancer. Nat. Rev. Urol..

[12] Ohta K., Ito A., Kuriyama S., Lupo G., Kosaka M., Ohnuma S., Nakagawa S., Tanaka H. (2011). Tsukushi functions as a Wnt signaling inhibitor by competing with Wnt2b for binding to transmembrane protein Frizzled4. Proc. Natl. Acad. Sci. U. S. A..

[13] Luehders K., Sasai N., Davaapil H., Kurosawa-Yoshida M., Hiura H., Brah T., Ohnuma S. (2015). The small leucine-rich repeat secreted protein Asporin induces eyes in Xenopus embryos through the IGF signalling pathway. Dev.

[14] Iozzo R.V., Buraschi S., Genua M., Xu S.Q., Solomides C.C., Peiper S.C., Gomella L.G., Owens R.C., Morrione A. (2011). Decorin antagonizes IGF receptor I (IGF-IR) function by interfering with IGF-IR activity and attenuating downstream signaling. J. Biol. Chem..

[15] Schönherr E., Sunderkötter C., Iozzo R.V., Schaefer L. (2005). Decorin, a novel player in the insulin-like growth factor system. J. Biol. Chem..

[16] Bengtsson E., Mörgelin M., Sasaki T., Timpl R., Heinegård D., Aspberg A. (2002). The leucine-rich repeat protein PRELP binds perlecan and collagens and may function as a basement membrane anchor. J. Biol. Chem..

[17] Lewis M. (2003). PRELP, collagen, and a theory of Hutchinson-Gilford progeria. Ageing Res. Rev..

[18] Bengtsson E., Aspberg A., Heinegård D., Sommarin Y., Spillmanni D. (2000). The amino-terminal part of PRELP binds to heparin and heparan sulfate. J. Biol. Chem..

[19] Happonen K.E., Fürst C.M., Saxne T., Heinegård D., Blom A.M. (2012). PRELP protein inhibits the formation of the complement membrane attack complex. J. Biol. Chem..

[20] Papadaki V., Asada K., Watson J.K., Tamura T., Leung A., Hopkins J., Dellett M., Sasaki N., Davaapil H., Nik-Zainal S., Longbottom R., Nakakido M., Torii R., Veerakumarasivam A., Kaneko S. (2020). Two secreted proteoglycans, activators of Urothelial cell–cell adhesion, negatively Contribute to bladder cancer initiation and progression. Cancers (Basel)..

[21] Rhodes D.R., Yu J., Shanker K., Deshpande N., Varambally R., Ghosh D., Barrette T., Pandey A., Chinnaiyan A.M. (2004). ONCOMINE: A cancer Microarray database and Integrated data-Mining Platform. Neoplasia.

[22] Hong R., Gu J., Niu G., Hu Z., Zhang X., Song T., Han S., Hong L., Ke C. (2020). PRELP has prognostic value and regulates cell proliferation and migration in hepatocellular carcinoma. J. Cancer.

[23] Rucci N., Capulli M., Ventura L., Angelucci A., Peruzzi B., Tillgren V., Muraca M., Teti A. (2013). Proline/arginine-rich end leucine-rich repeat protein N-terminus is a novel osteoclast antagonist that counteracts bone loss. J. Bone Miner. Res..

[24] Apweiler R. (2004). UniProt: The Universal protein knowledgebase. Nucleic Acids Res..

[25] Urner L.H., Liko I., Yen H.Y., Hoi K.K., Bolla J.R., Gault J., Almeida F.G., Schweder M.P., Shutin D., Ehrmann S., Haag R., Robinson C.V., Pagel K. (2020). Modular detergents tailor the purification and structural analysis of membrane proteins including G-protein coupled receptors. Nat. Commun..

[26] Werner H., Sarfstein R., Bruchim I. (2019). Investigational IGF1R inhibitors in early stage clinical trials for cancer therapy. Expert Opin. Investig. Drugs.

[27] Hakuno F., Takahashi S.I. (2018). 40 years of IGF1: IGF1 receptor signaling pathways. J. Mol. Endocrinol..

[28] Fürstenberger G., Senn H.J. (2002). Insulin-like growth factors and cancer. Lancet Oncol..

[29] Pollak M. (2008). Insulin and insulin-like growth factor signalling in neoplasia. Nat. Rev. Cancer.

[30] Kavran J.M., McCabe J.M., Byrne P.O., Connacher M.K., Wang Z., Ramek A., Sarabipour S., Shan Y., Shaw D.E., Hristova K., Cole P.A., Leahy D.J. (2014). How IGF-1 activates its receptor. Elife.

[31] Chen H.X., Sharon E. (2013). IGF-1R as an anti-cancer target-trials and tribulation. Chin. J. Cancer.

[32] Becker K., Cana A., Baumgärtner W., Spitzbarth I. (2018). p75 neurotrophin receptor: A Double-Edged Sword in Pathology and regeneration of the central nervous system. Vet. Pathol..

[33] Bothwell M. (2019). Recent Advances in Understanding Context-Dependent Mechanisms Controlling Neurotrophin Signaling and Function. F1000Res.

[34] Huang E.J., Reichardt L.F. (2001). Neurotrophins: Roles in neuronal development and function. Annu. Rev. Neurosci..

[35] Descamps S., Toillon R.A., Adriaenssens E., Pawlowski V., Cool S.M., Nurcombe V., Le Bourhis X., Boilly B., Peyrat J.P., Hondermarck H. (2001). Nerve growth factor Stimulates proliferation and survival of human breast cancer cells through two Distinct signaling pathways. J. Biol. Chem..

[36] Molloy N.H., Read D.E., Gorman A.M. (2011). Nerve growth factor in cancer cell death and survival. Cancers (Basel).

[37] Adriaenssens E., Vanhecke E., Saule P., Mougel A., Page A., Romon R., Nurcombe V., Le Bourhis X., Hondermarck H. (2008). Nerve growth factor is a potential therapeutic target in breast cancer. Cancer Res..

[38] Brandan E., Retamal C., Cabello-Verrugio C., Marzolo M.P. (2006). The low density lipoprotein receptor-related protein functions as an endocytic receptor for decorin. J. Biol. Chem..

[39] Cabello-Verrugio C., Santander C., Cofré C., Acuña M.J., Melo F., Brandan E. (2012). The internal region leucine-rich repeat 6 of decorin interacts with low density lipoprotein receptor-related protein-1, modulates Transforming Growth Factor (TGF)-β-dependent signaling, and inhibits TGF-β-dependent fibrotic response in skeletal muscles. J. Biol. Chem..

[40] Zilberberg A., Yaniv A., Gazit A. (2004). The low density lipoprotein receptor-1, LRP1, interacts with the human Frizzled-1 (HFz1) and down-regulates the canonical Wnt signaling pathway. J. Biol. Chem..

[41] Hayashi S., Takeichi M. (2015). Emerging roles of protocadherins: From self-avoidance to enhancement of motility. J. Cell Sci..

[42] Kim S.Y., Yasuda S., Tanaka H., Yamagata K., Kim H. (2011). Non-clustered protocadherin. Cell Adhes. Migr..

[43] Langenhan T., Aust G., Hamann J. (2013). Sticky signaling-Adhesion class G protein-coupled receptors take the stage. Sci. Signal..

[44] Paavola K.J., Hall R.A. (2012). Adhesion G protein-coupled receptors: Signaling, pharmacology, and mechanisms of activation. Mol. Pharmacol..

[45] Bengtsson E., Lindblom K., Tillgren V., Aspberg A. (2016). The leucine-rich repeat protein PRELP binds fibroblast cell-surface proteoglycans and enhances focal adhesion formation. Biochem. J..

[46] Rucci N., Rufo A., Alamanou M., Capulli M., Del Fattore A., Åhrman E., Capece D., Iansante V., Zazzeroni F., Alesse E., Heinegård D., Teti A. (2009). The glycosaminoglycan-binding domain of PRELP acts as a cell type-specific NF-κB inhibitor that impairs osteoclastogenesis. J. Cell Biol..

[47] Botos I., Segal D.M., Davies D.R. (2011). The structural biology of Toll-like receptors. Structure.

[48] Kobe B., Kajava A.V. (2001). The leucine-rich repeat as a protein recognition motif. Curr. Opin. Struct. Biol..

[49] Xie H., Griffin T.J. (2006). Trade-off between high sensitivity and increased potential for false positive peptide sequence matches using a two-dimensional linear ion trap for tandem mass spectrometry-based proteomics. J. Proteome Res..

[50] Scalf M., Westphall M.S., Smith L.M. (2000). Charge reduction electrospray mass spectrometry. Anal. Chem..

[51] Hopper J.T.S., Sokratous K., Oldham N.J. (2012). Charge state and adduct reduction in electrospray ionization-mass spectrometry using solvent vapor exposure. Anal. Biochem..

[52] Kawamura T., Nomura M., Tojo H., Fujii K., Hamasaki H., Mikami S., Bando Y., Kato H., Nishimura T. (2010). Proteomic analysis of laser-microdissected paraffin-embedded tissues: (1) Stage-related protein candidates upon non-metastatic lung adenocarcinoma. J. Proteomics.

[53] Keller A., Nesvizhskii A.I., Kolker E., Aebersold R. (2002). Empirical statistical model to estimate the accuracy of peptide identifications made by MS/MS and database search. Anal. Chem..

[54] Nesvizhskii A.I., Keller A., Kolker E., Aebersold R. (2003). A statistical model for identifying proteins by tandem mass spectrometry. Anal. Chem..

[55] Ashburner M., Ball C.A., Blake J.A., Botstein D., Butler H., Cherry J.M., Davis A.P., Dolinski K., Dwight S.S., Eppig J.T., Harris M.A., Hill D.P., Issel-Tarver L., Kasarskis A., Lewis S. (2000). Gene ontology: Tool for the unification of biology. Nat. Genet..

